# Regulation of T3 Availability in the Developing Brain: The Mouse Genetics Contribution

**DOI:** 10.3389/fendo.2018.00265

**Published:** 2018-05-28

**Authors:** Sabine Richard, Frédéric Flamant

**Affiliations:** Institut de Génomique Fonctionnelle de Lyon, INRA USC 1370, Université de Lyon, Université Lyon 1, CNRS UMR 5242, Ecole Normale Supérieure de Lyon, Lyon, France

**Keywords:** thyroid hormones, neurodevelopment, neurodevelopmental disorder, placenta, brain–blood barrier, transporters

## Abstract

Alterations in maternal thyroid physiology may have deleterious consequences on the development of the fetal brain, but the underlying mechanisms remain elusive, hampering the development of appropriate therapeutic strategies. The present review sums up the contribution of genetically modified mouse models to this field. In particular, knocking out genes involved in thyroid hormone (TH) deiodination, transport, and storage has significantly improved the picture that we have of the economy of TH in the fetal brain and the underlying genetic program. These data pave the way for future studies to bridge the gap in knowledge between thyroid physiology and brain development.

## Introduction

The major role of thyroid hormone (TH) in brain development has been recognized for a long time, but, in spite of considerable progress in the understanding of TH mode of action, the underlying mechanisms remain elusive. The thyroid gland produces mainly thyroxine (T4), the major circulating form of TH. Deiodination ensures the conversion of T4 into 3,3′,5-triiodothyronine (T3), the active form of TH, in several organs. This enzymatic reaction can be catalyzed either by type 1 deiodinase (DIO1) in the liver or by type 2 deiodinase (DIO2) in several other organs including the brain. TH catabolism is ensured by DIO1 and type 3 deiodinase (DIO3). T3 exerts a pleiotropic influence on development and cell differentiation. it does so by binding to nuclear receptors (TRs, including the TRα1, TRβ1, and TRβ2 isotypes) present in most, if not all, cell types in all vertebrate species. The consequences on neurodevelopment of a deficit in TH, either during pregnancy or during early childhood, are mostly irreversible (1). However, our basic knowledge of TH function during fetal brain development remains limited. Meanwhile, the possibility that genetic alterations (2) or environmental contaminations (3) compromise TH signaling in the fetal brain, without necessarily modifying the circulating levels of TH, is a matter of concern. Moreover, when circulating TH levels are impacted, neither the quantitative relationships nor the timing between serum alterations and brain effects have been well characterized. This review focuses on the contribution of experimental genetics in mice to our basic understanding of the complex relationship that links maternal thyroid physiology to fetal neurodevelopment. Notably, the delivery of T3 to the fetal brain seems to be a highly controlled and complex mechanism, which recent genetic investigations have only begun to unravel.

## Time Course of TH Action During Human Brain Neurodevelopment

Thyroid hormone has for a long time been known to exert a major influence on human neurodevelopment, during prenatal and post-natal life. Three prenatal stages are to be considered (4):

(1) The first trimester of pregnancy is essentially dedicated to neurulation, brain patterning, and active neural cell proliferation. By the end of the second month, the rudimentary structures of the brain and central nervous system are established. In the earliest neurodevelopmental stages, TH is present at very low levels in the celomic and amniotic fluids, while TH levels are high in the maternal serum. TH levels increase rapidly in the celomic and amniotic fluids after the 4th week of pregnancy (5). The nuclear receptors of T3 are already present in the embryonic brain at this stage, and partially occupied by T3 (6). As a consequence, it is likely that gene expression can already be activated by T3 at an early stage of development. Indeed, TRs are seen very early in brain development, from 10 weeks of gestation in the human fetal brain (6) well before the fetal thyroid gland is functional. Thus, even minimally reduced maternal TH levels in early pregnancy may result in adverse outcome in the offspring (7). The possibility remains for T3 to be absent in some brain areas where TRs are expressed. In such cases, unliganded TRs would be bound to DNA and recruit corepressors, repressing the expression of the TH-inducible genes.
(2) The second trimester is marked by an acceleration in brain expansion and by massive neuronal and glial cell differentiation. The amniotic cavity grows rapidly and the matured placental barrier controls the passage of biological material from maternal to fetal circulation. While the development of the thyroid gland starts within the first weeks of pregnancy, the capacity of thyrocytes to accumulate iodine appears only at the end of the 4th month, and the secretion of TH by the fetal thyroid gland starts even later. Therefore, maternal TH remains, at mid-gestation, the main supply of TH for the fetus. Although TH concentration remains low in the fetal serum, this concentration increases rapidly in the cortex from the beginning of the second trimester. Thus, the concentration of T3 in the fetal cortex eventually reaches or even exceeds adult values (8). Concomitantly, TR levels in the fetal brain peak around the 16th week, in coincidence with the period of neuroblast multiplication.
(3) During the third trimester, neurodevelopment is clearly asynchronous, and rapid expansion is restricted to a few brain areas, notably the cerebellum and the olfactory bulbs. In other brain areas, terminal maturation processes, axon myelination, and synapse formation predominate. The fetal thyroid gland is functional but the complete maturation of the hypothalamus–pituitary–thyroid gland axis will only be achieved within the last month of pregnancy. Thus, a significant fraction of TH in the fetus is still of maternal origin (9). The levels of T4 and T3 in the fetal serum progressively reach levels found in newborns.

## Consequences of TH Deficiency on Human Neurodevelopment

Overall, the risk of presenting neurodevelopmental and psychiatric disorders due to maternal thyroid dysfunction, as well as the nature and severity of the symptoms, depend on two parameters: the time window and the severity of TH deficiency (10, 11). While overt maternal hypothyroidism during pregnancy causes a condition known as “neurological cretinism”—with most dramatic effects if hypothyroidism occurs during the first trimester (12–14), only large epidemiological studies can provide evidence for cognitive defects caused by mild alterations in maternal TH levels taking place during late pregnancy (15). Importantly, therapeutic interventions are risky, because prolonged exposure to excess of TH during pregnancy increases the risk of miscarriage and may also probably cause later cognitive and psychiatric disorders (15, 16).

## The Mouse as a Model of Human Pregnancy

**Figure 1:**
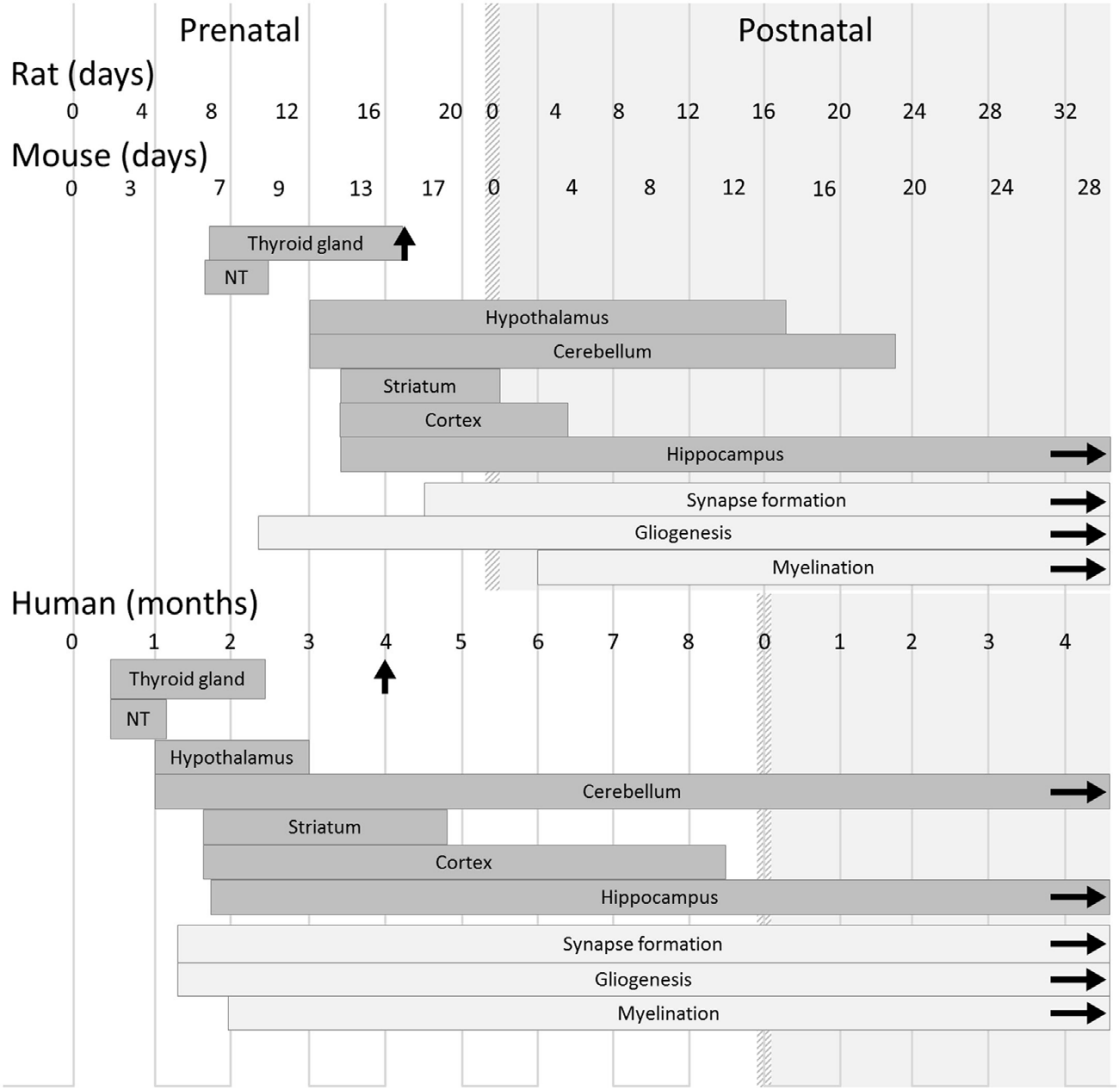
Timeline of thyroid and brain development in humans and rodents. In both rodents and humans, the development of the main brain areas starts before the onset of the fetal thyroid gland function and relies on maternal thyroid hormone supply. However, differences between species must be kept in mind when translating results from rodent models to humans, and even between mouse and rat models. Gestation lasts 22 days in rats, 19 days in mice, and 42 weeks in humans. Gliogenesis, myelination, and synaptogenesis are asynchronous in different brain areas and continue after birth. For each brain region, the corresponding rectangle symbolizes the phase of neurogenesis. Vertical arrows indicate the initiation of iodide uptake by thyrocytes, which marks the end of thyroid gland maturation. The light gray area corresponds to postnatal life. Abbreviation: NT, neural tube [adapted from Ref. (19)].

Although classical animal studies of TH signaling during gestation have been mainly done in the rat, the mouse is the favorite model for experimental genetics, due to the possibility of generating knock-out (KO) and knock-in mutations. Most of the effects of hypothyroidism that were described in the rat are also found in the mouse. Nevertheless, the differences between the two rodent models require minor adjustments (Figure 1). For example, gestation lasts for 22 days in rats and 19 days in mice, but the stage at which the thyroid gland is functional is similar in both species (around gestational day 17; GD17). As a consequence, the mouse fetus remains dependent on maternal TH up to later stages of *in utero* development than does the rat fetus (about 89% of the duration of gestation in mice *vs* about 77% of the duration of gestation in rats) (17). Accordingly, differences between the two species as regards the consequences of hypothyroidism on neurodevelopment have been reported. For example, a malformation of the corpus callosum, with abnormal presence of neurons in the white matter, has been observed in hypothyroid rats but not yet been reported in mice (18).

By contrast, notable differences in the program of neurodevelopment should be carefully taken into account when translating rodent studies to humans (Figure 1) (19). The onset of thyroid gland function in rodents occurs at a late stage of gestation (GD17), and the fetus largely relies on TH of maternal origin, whereas in humans, the thyroid gland becomes functional during the 4th month of gestation. In rodents as in humans, the first third of gestation appears to be of critical importance for the action of TH on neurodevelopment (20). However, at birth, neurodevelopment is less advanced in mice than in humans. Markedly, cerebellum expansion, axon myelination, and synapse formation, which take place during the last trimester of human pregnancy (21), are mainly post-natal events in mice as in rats. Interestingly, this post-natal phase in rodents is marked by a peak in circulating levels of TH (22). It is unclear whether an equivalent peak in TH levels occurs at any point during brain development in humans. As a whole, the mouse post-natal development is, to some extent, relevant to human late pregnancy. Moreover, a recent study has provided evidence that in mice, as in rats and in humans, mild hypothyroidism in the perinatal period is sufficient to cause long-lasting cognitive impairments that persist into adulthood (23).

Brain cells of rodent fetuses are sensitive to TH and already express *Thra*, the gene encoding TRα1, the most ubiquitous nuclear receptor of TH. Early indirect evidence, based on binding affinity measurements, suggested that TRβ1, rather than TRα1, was the first receptor to be present in human fetal brain (6). However, recent transcriptome data (http://www.brainspan.org) indicate that, like in rodents (24, 25), *Thra* expression in the human fetal brain is ubiquitous and precocious, while *Thrb* expression is restricted to specific brain areas and appears at a later stage. This conclusion is also consistent with the fact that neurodevelopmental defects seem to be more frequent in patients with *Thra* mutations (26), than in patients with *Thrb* mutations (27). Like TRβ1, TRα1 can exert both ligand-dependent and ligand-independent influence on gene expression. While *Thra* KO mice lose both activities, knock-in mutations maintain ligand-independent repression. Therefore, *Thra* knock-in models display severe neurodevelopmental defects, mimicking early TH deficiency, which are not observed in KOs (28, 29). The contribution of mouse genetics to the understanding of *Thra* and *Thrb* neurodevelopmental functions has been recently reviewed elsewhere (30).

## Neurodevelopmental Consequences of Congenital Hypothyroidism in Rodents

Congenital hypothyroidism is experimentally induced in rodents by reducing thyroid signaling, by exposure to a toxic substance, surgical thyroidectomy, or genetic mutation. Congenital hypothyroidism seems to affect all the main neural cell types in all brain areas in rodents. Many studies have been focused on the cortex and cerebellum, pointing to similarities between the two brain areas, even though they develop during different time windows (1). These similarities suggest the existence of common mechanisms. Studying the two models, cortex and cerebellum, should highlight unifying principles regarding the role of TH in neurodevelopment.

One major defect observed in the hypothyroid brain is that axon myelination is retarded, due to impaired differentiation of oligodendrocytes (31). Another typical feature is delayed maturation of radial glia that act as a scaffold for neuron migration during corticogenesis. Accordingly, the radial migration of glutamatergic neuron progenitors is impaired (32, 33). The migration of GABAergic interneuron progenitors follows different routes but is also disrupted (34, 35). In addition, the terminal differentiation of these inhibitory neurons is deeply altered (34, 36). Cell type-specific blockade of TRα1 function in the cerebellum has led to the conclusion that, during neurodevelopment, the primary targets of T3 are the GABAergic neurons and radial glia (37). These cell types interact with the other neighboring cell types, either by direct contact or by exchanging growth factors and neurotrophins (38). Therefore, while the initial cell-autonomous action of T3 appears to be restricted to few cell types, it is subsequently amplified by these T3-induced secretions. As a consequence, T3 indirectly influences the differentiation of all neural cell types (39). Although the molecular mechanisms underlying all these cellular defects remain largely unknown, genome-wide expression analyses have identified a number of TH responsive genes (40). In particular, *Hr* (encoding Hairless) and *Klf9* (encoding Kruppel-like factor 9 protein) are highly responsive to T3 in several brain areas and cell types and are routinely used as intracerebral sensors of T3 signaling (41, 42).

## The Origin of T3 in the Fetal Rodent Brain

Even after the onset of the fetal thyroid gland function, 60% of the total TH content in fetal peripheral tissues remains of maternal origin at late gestation stages in mice (43). In addition, there are good indications supporting the view that the main source of T3 in the fetal brain is maternal T4, not maternal T3. Indeed, when hypothyroid dams are given either T4 or T3 during gestation, T4 is more efficient at restoring T3 level in the fetal cortex (44). Moreover, maternal hypothyroxinemia (i.e., the selective reduction of T4, but not T3, in the maternal serum) causes neurodevelopmental defects in rats similar to maternal hypothyroidism. Therefore, maternal T3 is not sufficient to ensure correct fetal neurodevelopment (45, 46). Such a predominance of maternal T4 over maternal T3 in supplying THs to the fetal brain results from the existence of two barriers, first at the placenta level and second at the blood–brain barrier level, which display some selectivity for T4 or T3.

Maternal T4 and T3 are catabolized in the placenta by DIO3 (47), which has a preference for T3 over T4 as a substrate (48). As the concentration of T4 in the maternal serum exceeds that of T3, the net result is a preferential transfer of maternal T4 to the fetal serum (49). Furthermore, during fetal development, the contribution of fetal circulating T3 to brain T3 appears to be very low, suggesting that T3 is unable to cross the blood–brain barrier at this early stage (43). The collective term “blood–brain barrier” encompasses three putative pathways for TH entry in the brain: the blood–brain barrier proper, the blood–cerebrospinal fluid barrier, and the cerebrospinal fluid–brain barrier. Most of T3 in the fetal brain comes from T4 transport across the blood–cerebrospinal fluid barrier (i.e., the choroid plexus). T4 is locally converted into T3 by DIO2, which is expressed in the brain ventricles as early as GD15 (50). By contrast, after birth, the fraction of brain T3 directly coming from the circulation reaches 50% (43).

Finally, specific mechanisms come into play to ensure an accumulation of T3 in the fetal brain. Indeed, at mid-gestation, the level of T3 in the fetal brain is higher than in the fetal serum or liver (51). Under low levels of maternal T4, fetal T4 levels are decreased in peripheral organs and in the brain, but brain T3 level remains unchanged (43).

## Heterogeneity of the Intracerebral Distribution of T3

Quantitative analysis of TH content in small brain areas by biochemical means is technically challenging. To circumvent this problem, transgenic models have been developed to indirectly evaluate T3 signaling. *FINDT3* transgenic mice carry a self-inducing reporter construct based on an artificial Gal4-TRα1 receptor (50). In this system, the regulation of *lacZ* expression allows one to directly visualize TH signaling in the fetal brain. X-gal staining, to visualize *lacZ* expression, confirms the entry of maternal TH in the fetal mouse brain at mid gestation. Two simpler, but less specific, transgenic devices have also been produced: *TRE2x* mice carry the *lacZ* gene, the expression of which is driven by a minimal promoter and two binding sites for endogenous TR (52). *THAI* mice design followed a similar strategy, but the luciferase gene was preferred to *lacZ* to facilitate quantification and live imaging (53). The *lacZ* reporter expression patterns in *FINDT3* and *TRE2x* mice do not always match. Although this is expected, as they rely on very different systems, the presence of experimental biases cannot be completely ruled out, as both systems could be influenced by other factors than T3 local concentration. For example, TRE2x is also regulated by other nuclear receptors recognizing the same response element, notably LXR (53). Meanwhile, the minimal promoter used in FINDT3 mice favors the expression in neurons but is inefficient in glia (50). In spite of these differences, both *FINDT3* and *TRE2x* transgenic models detect the presence of T3 in the fetal brain at mid-gestation (GD12 for *TRE2x*, GD13 for *FINDT3*). Both models also converge to suggest a persistent heterogeneity of T3 distribution in the brain. The detailed expression pattern of *lacZ* in *FINDT3* mice during fetal brain development has been reported elsewhere (50). In summary, the reporter expression is high in the midbrain, anterior cortex and hippocampus but low in the cerebellum and olfactory bulbs throughout late fetal and early post-natal development. This expression pattern is maintained in the adult brain and fits with direct measurement of T3 content in different rat brain areas (54). This expression pattern also presents similarities with the situation in humans, where T3 content is high in the fetal cortex and low in the fetal cerebellum (8). While the existence of such heterogeneous distribution of T3 in the brain is confirmed by different approaches, the underlying mechanisms remain unclear.

## Deiodinases and Brain T3 Content

As fetal T3 is mainly produced by DIO2-mediated T4 deiodination, the heterogeneous distribution of T3 in the fetal brain has been hypothesized to be a consequence of the heterogeneous distribution of DIO2 in the brain (50, 55). However, there is, to our knowledge, no genetic evidence that DIO2 is necessary to maintain the level of T3 in the fetal brain. Indeed, *Dio2* gene KO in mice does not modify the *FINDT3* reporter expression pattern throughout gestation (unpublished data). Even the combination of *Dio1/Dio2* KO does not induce major changes in the T3 content of different brain areas, as measured at post-natal day 6 (56). In *Dio1/Dio2* KO mice, T3 content is not significantly affected until post-natal day 15 (PND15). From that stage onward, T3 levels are significantly reduced in all *Dio1/Dio2* KO mouse brain areas (57). However, T3 signaling in neural cells is not immediately affected, as expression of the *Hairless* gene at PND21 is not changed. As mentioned above, this gene is a well-characterized T3-responsive gene and is often used as an internal sensor of T3 signaling in the brain (58). Taken together, these data seem to indicate that, in mice, T3 levels in the brain do not rely on type 2 deiodination before the second week of post-natal life.

The surprisingly limited consequences of *Dio2* KO on fetal neurodevelopment might reflect the intervention of compensatory mechanisms in the brain. In particular, the expression of the *Dio3* gene, encoding DIO3, which is responsible for TH catabolism in several tissues, is reduced in the brain of *Dio2* KO mice, therefore limiting TH deficiency (17). As a whole, it remains unlikely that DIO2 plays a major role in generating a heterogeneous distribution of T3 in the brain. An alternative hypothesis to the local conversion of T4 into T3 would be a selective catabolism in different brain areas. This possibility was tested by crossing FINDT3 reporter mice with *Dio3* KO mice. In *FINDT3/Dio3* KO mice, the reporter gene senses an increase in TH signaling in several brain areas and *lacZ* expression is higher than in *FINDT3* mice. However, this difference occurs only weeks after birth, and the expression pattern of the reporter is not markedly altered (59). Therefore, local metabolism by deiodinases has the capacity to buffer variations in TH availability but cannot alone account for the heterogeneous distribution of T3 in fetal and post-natal mouse brains.

## TH Transporters

### TH Transport: Many Players

If the accumulation and heterogeneous distribution of T3 in the fetal brain are not only the result of local TH metabolism, what could be the underlying mechanisms? An alternative could be that TH transport has a major influence.

It is now recognized that specific transporters are needed for TH to cross the placenta, the blood–brain barrier or the blood–cerebrospinal fluid barrier (43, 60). Intracytoplasmic transporters are also needed for T3 to reach cell nuclei (61). While solute carrier proteins are supposed to be key elements of TH transfer to the fetal brain, progress of genetic investigation in this field has been slow for several reasons. First, THs can be transported with variable efficiency by at least 20 members of the solute carrier family (62). Many of these are present in the brain, which suggests the existence of several entry routes with redundant functions. Therefore, a single gene KO may not be sufficient to alter TH transfer. The second difficulty is that some transporters are expressed in both the placenta and the fetal brain, which might introduce a confounding effect: neurodevelopmental defects due to placental defect might be mistakenly attributed to a direct influence of the KO in the brain (63). Finally, these transporters are often able to transport other low molecular-weight molecules that are also required for proper neurodevelopment. One such example is that of the L-type amino-acid transporter type 1 (LAT1), which is known to facilitate TH cellular uptake (64). The KO of *Slc7a5*, which encodes LAT1, has clear neurological consequences, even when the KO is restricted to the blood–brain barrier. However, the effects are not attributed to TH deficiency, but to a defect in neutral amino-acid transport, since intra-cerebro-ventricular injection of amino-acids provides a significant improvement in the neurobehavioral abnormalities caused by this mutation (65).

### The Intriguing Case of Mct8

Among all solute carrier proteins, MCT8 deserves special attention, for the following reasons: (a) T4, T3, and reverse T3 are the only known substrates of this transporter (66). (b) *Mct8* gene expression in the mouse brain starts at embryonic day 15 (67). Its expression is notably high at post-natal stages in the cortex, striatum, hippocampus, and in the tanycytes lining the hypothalamic third ventricle (68). (c) Human *Mct8* gene mutations cause a dramatic neurodevelopmental disorder known as the Allan–Herndon–Dudley syndrome (69). Surprisingly, *Mct8* KO in mice has only minor neurodevelopmental consequences (70, 71).

The expression pattern of the TH signaling reporter is not visibly changed in the brain of *FINDT3/Mct8* KO mice, arguing against a predominant influence of this transporter in mice (unpublished data). Unexpectedly, a transient increase in cortical T3 content is observed between PND0 to PND3 in *Mct8* KO mice (72), while the serum content of T4 is high and of T3 is low. The situation is reversed at PND21, when T3 is reduced in the cortex and high in the plasma of *Mct8* KO mice. Despite a chronic excess of T4 and T3 in the serum, the adult brain remains hypothyroid (73). *In vivo* transport of T3, but not T4, is markedly reduced in the brain (71). Taken together, these data suggest that, in mice, the predominance of MCT8 function as a TH transporter in the brain only appears at a late post-natal stage. At this point, *Mct8* KO mice start to display a clear deficit in intracerebral T3 content. Interestingly, gene expression analyses also suggest that *Mct8* KO selectively affects some brain areas, notably the hypothalamus, but not others, like the striatum (71).

There are two possible explanations for the discrepancy between the severity of the Allan–Herndon–Dudley syndrome and the modest consequences of *Mct8* KO in mice: one is that human MCT8, but not mouse MCT8, is necessary to transport some unknown molecule required for neurodevelopment. This might explain why the consequences of the Allan–Herndon–Dudley syndrome are usually much more severe than the symptoms of congenital hypothyroidism. Alternatively, due to their different spatial and temporal patterns of gene expression, compensatory mechanisms might exist in mice which cannot take place in humans (74).

### Combining Gene KOs to Reveal Mct8 Function

A number of combinations of KOs have been tested to identify another transporter able to compensate for the absence of MCT8 in mice, but not in humans (Table 1). MCT10 transporter is absent at the blood–brain barrier and, accordingly, the combination of *Mct10* and *Mct8* KOs does not aggravate the *Mct8* KO brain phenotype (68). Combining *Mct8* and *Lat2* KOs has also failed to demonstrate a major function of LAT2 in TH transport in the brain (72). OATP1C1 is a better candidate, as it is encoded by a gene that is apparently expressed in the mouse fetal brain at higher level than in the human fetal brain. As expected, the combination of both *Oatp1c1* and *Mct8* KOs markedly increase the post-natal neurodevelopmental defects of single KO, despite a compensatory increase in *Dio2* expression (75). Notably, while the reduction of *Hairless* expression in the cortex (PND 21) is moderate after a single KO, the reduction in gene expression achieved when the two mutations are combined is comparable to the one observed in TH-deficient pups (75). A similar aggravation of the phenotype is observed for axon myelination in the corpus callosum and for GABAergic neuron differentiation in the cortex (75). All these data point to a state of severe hypothyroidism in the brain of *Mct8/Oatp1c1* mice at PND21.

**Table 1:** Neurodevelopmental consequences of knock-out (KO) mutations in thyroid hormone (TH) transporters and storage proteins.

Gene name (official name)	Reference	Neurodevelopmental and other brain phenotype
**Single KO**		
*Crym*	(76)	Rapid turnover of T3 in the adult brain
*Dio2*	(17, 56, 77, 78)	Reduced expression of TH target genes (*Hr* and *Shh*) at PND3. Reduced T3 content in the cerebellum and hippocampus at post-natal day 15. Increased DIO3 activity in the adult brain
*Dio3*	(59, 79, 80)	Local accumulation of T3 in specific brain areas during post-natal life. Enhanced response to T3 at PND21
*Lat1 (Slc17a5)*	(65)	Motor delay and autism-related phenotypes. Altered function of GABAergic neurons
*Lat2 (Slc7a8)*	(72, 81)	Slight impairment in adult motor coordination. Slight reduction of T3 level in the post-natal brain and serum (PND5 and PND21)
*Mct8 (Scl16a2)*	(70–72)	Transient increase in T3 level in the post-natal brain (PND0 to 5), followed by persistent T3 deficiency in the brain. Increased DIO2 activity in the adult brain. Low T3 level in late post-natal and adult brains
*Mct10 (Slc16a10)*	(82)	No adverse effect observed
*Oatp1c1 (Slc1c1)*	(83)	Moderate decrease in T4 and T3 brain content (PND21)
*Oatp4a1 (Slc4a1)*	(84)	ND
*Oatp1a4 (Slc1a4)*	(85)	ND
*Ttr*	(86, 87)	Low T4 and T3 level in the choroid plexus in the adult brain. Normal levels in the cortex, hippocampus, and cerebellum. No change in DIO2 activity

**Multiple KOs**		
*Dio2* + *Dio3* or *Dio1* + *Dio2* + *Dio3*	(56)	Increased T3 content in the adult brain
*Mct8* + *Lat2*	(72)	Transient decrease in T3 content in the brain after birth compared to *Mct8* KO
*Mct8* + *Dio2*	(58, 88)	Decreased level of brain T3 signaling at PND21 and in adults (more pronounced than in *Mct8* KO)
*Mct8* + *Dio3*	(89)	Level of brain T3 signaling intermediate between *Mct8* KO and *Dio3* KO (PND2 to adult)
*Mct8* + *Oatp1c1*	(75)	Hypothyroid cortex, hippocampus, and striatum at PND21. Impaired myelination and GABAergic differentiation. Low T4 uptake in the adult brain
*Mct8* + *Mct10*	(68)	Increased level in serum T4 induces an increase in T4 and T3 content in the adult brain

Interestingly, *Hairless* gene expression can be restored in the brain of hypothyroid wild-type mice by either T4 or T3 treatment. By contrast, when *Mct8* KO mice are made hypothyroid by pharmacological means, only T4 treatment is able to restore *Hairless* gene expression in the brain (90), suggesting that in the absence of MCT8, deiodination of T4 into T3 becomes mandatory for T3 accumulation in the brain. In agreement with this hypothesis, the combination of *Dio2* and *Mct8* KOs also alters post-natal neurodevelopment more visibly than either single KO, as judged by gene expression analysis at PND21 (58). The synergy of these two KOs indicates the existence of two independent pathways for T3 entry in the brain. *Dio2* and *Mct8* are thus key elements of these two pathways ensuring the presence of T3 in neurons (91).

## Intracerebral Storage of T3

At least some of the solute carriers that transport TH can act both on cellular influx and efflux (92). Therefore, the maintenance of sustained TH levels in the brain, including when circulating levels are low, might not rely exclusively on transporters. The accumulation of TH in the fetal brain implies the presence of other TH binding proteins for storage, either in neural cells or in the cerebrospinal fluid. One of the main TH storage proteins is transthyretin, which is encoded by the *Ttr* gene. Transthyretin is present in the serum, placenta and is abundant in the cerebrospinal fluid. While *Ttr* gene expression is already high at mid-gestation in the choroid plexus, *Ttr* KO has no visible neurodevelopmental consequences (86). Indirect evidence also indicates that brain TH levels are not altered in *Ttr* KO mice (93).

Another molecule contributing to TH accumulation in the fetal brain is CRYM (alias μ-crystallin and CTBP), which is a cytosol protein that binds TH in a NADPH-dependent manner. According to the Allen Brain Atlas, *Crym* gene is already expressed at mid-gestation in the fetal mouse brain. *Crym* expression is high in some brain areas, notably in the cortex, and its expression pattern changes rapidly during development. Once more, *Crym* KO has no obvious adverse effect on neurodevelopment. However, the clearance of radiolabeled T3 from the brain is accelerated in adult *Crym* KO, compared to wild-type, mice (76). Originally considered exclusively as a cytoplasmic storage protein, CRYM was also later identified as an enzyme that reduces the sulfur-containing cyclic ketimines (94), which are potential neurotransmitters. Structural data suggest that T3 is an allosteric regulator that inhibits CRYM enzymatic activity (95). Thus, the regulation of CRYM enzymatic activity by T3 might constitute a way by which T3 influences brain function without binding to its nuclear receptors.

## What is the Function of T3 During Fetal Brain Development?

While a number of transcriptome analyses have been performed at post-natal stages in order to identify T3-responsive genes in the post-natal brain (40), little information is available for the fetal brain. Microarray analyses have identified a large number of genes, the expression of which is sensitive to maternal hypothyroidism in rat cortex and hippocampus (96, 97). However, the changes in gene expression observed in these studies might be very indirect consequences of hypothyroidism. Indeed, some of these changes in gene expression may reflect metabolic changes, defects in placenta development, or a modification in the composition of the heterogeneous population of neural cells. Understanding the mechanisms of action of T3 in the fetal brain requires separating T3 direct target genes from genes that are positioned downstream in the cascade of effects induced by hypothyroidism.

A more direct approach to recognize T3-responsive genes was to first submit pregnant mice to a short methimazole treatment to make them hypothyroid and then treat them with THs for 12 h (98). Such a short-term activation of TH signaling pathways reduces the chances of observing indirect effects because it allows little time for activation of secondary target genes, i.e., genes that are activated by proteins encoded by direct T3 target genes. This approach confirmed the existence of a large number of TH-responsive genes in the fetal cortex, although discrepancies between studies were also noticed. These genes produce both messenger RNAs and microRNAs (98). Primary cell cultures from mouse fetal cortices, enriched in either neurons or astrocytes, were also used. When analyzed at a genome-wide scale, the *in vitro* response to T3 confirmed the existence of many T3-responsive genes in both cell populations. However, none of the above-mentioned protocols allows eliminating completely all secondary effects of TH on gene expression. Thus, the observed changes in gene expression can still either be directly mediated by TRα1 and TRβ1 or be secondary to the initial response of the cells, which consists in overexpressing a number of transcription factors and growth factors.

To distinguish between direct and indirect T3 target genes, cycloheximide was used to inhibit protein synthesis and to prevent secondary responses. Among the previously identified T3-responsive genes, a subset of 371 genes was still responsive in the presence of cycloheximide. This smaller set of genes was likely to contain those that are directly regulated by TRα1 (99, 100). In this *in vitro* system, T3 response was, at least for a large part, similar to the one observed in more physiological settings (40). Interestingly, in-depth bioinformatics analysis led to the hypothesis that some unidentified cortical cell types displayed a higher sensitivity to T3 than the others (99, 100). However, in that study, it was difficult to ascertain a direct transcriptional response, since there was no knowledge about the genomic sites occupied by TRα1 and TRβ1 in the cells used.

The number of genes that are regulated in neural cells, the variety of their function, and some discrepancies between published studies do not allow for a unified interpretation of these transcriptome data. It is nevertheless striking that T3 activates the expression of genes involved in several signaling pathways known to be important for neurodevelopment, such as the Sonic hedgehog, glucocorticoid, and retinoic acid signaling pathways (99). These regulations suggest that, in the fetal cortex, as in the post-natal cerebellum, the initial response to T3 of a subset of cell types is greatly amplified by the activation of other signaling pathways (37, 101).

## Trans-Generational Maternal Transmission of Hypothalamus–Pituitary–Thyroid Axis Set Points?

Only a few studies have to date addressed the capacity of TH to promote hypothalamus development (102). However, one important consequence of a prenatal change in TH signaling is a permanent change in the set points of the hypothalamo–pituitary–thyroid gland axis in the progeny (103). This might reflect, at least in part, a defect in hypothalamus development, perhaps in the development of hypophysiotropic neurons which produce thyrotropin-releasing hormone. For example, mice born from *Thrb* KO dams are exposed to an excess of TH during fetal life. When they reach adulthood, their circulating level of thyroid-stimulating hormone is lower than in control mice born from euthyroid dams. Despite nearly normal levels of T4 and T3, *Dio2* gene expression in the hypothalamus is reduced and *Dio3* gene expression in the pituitary is augmented (104, 105). Thus, there is a theoretical possibility for intergenerational and transgenerational maternal transmission of changes in TH signaling acquired during fetal life.

## Future Directions and Clinical Relevance

Taken together, these genetic data pave the way for promising studies. One clear gap in our knowledge is the development and physiology of the barriers that TH must cross to reach neural cells and the possible involvement of TH itself in these processes (11). Currently, mouse genetics do not bring much information to our understanding of either the action of TH on placenta development (106) or the mechanisms of trans-placental transfer of TH (107). However, there is a possibility that a placental defect, of maternal or fetal origin, may indirectly compromise neurodevelopment, as observed in other contexts (108, 109). The placental barrier displays some similarities with the blood–brain barrier and also expresses a number of solute carrier family members (92, 110). In fact, placental development in *Mct8* KO fetuses is slightly altered (111). Another limitation of current studies is that TH entry in the fetal brain has been mainly considered in a global manner. However, the existence of different pathways for TH entry in the brain is an indication that T3 can be delivered to specific brain areas at different times during neurodevelopment. This may allow the local concentration of T3 to act as a positional and temporal cue for neural cell differentiation.

Our understanding of the molecular mechanisms underlying the influence of T3 in neuronal migration and differentiation also remains very limited and should be integrated in the general model of neurodevelopment that has emerged from a large body of mouse genetic studies (112). The recent advent of “omics” has not yet provided an unbiased and genome-wide view of T3 influence on gene expression, within each of the many cell types that are present in the developing brain.

Improving our understanding of these mechanisms should be beneficial in several respects. First, it may help to understand the pathology of rare genetic diseases altering cognitive function. Also, it should help to better address the neurotoxicity of environmental chemicals acting as TH disruptors. Finally, it could lead to the development of new TH analogs suitable to treat neurodevelopmental and psychiatric diseases.

## Author Contributions

FF conceived and wrote the article. SR wrote the article.

## Conflict of Interest Statement

The authors declare that the research was conducted in the absence of any commercial or financial relationships that could be construed as a potential conflict of interest.
